# Dose-efficient phase-contrast imaging of thick weak phase objects via OBF STEM using a pixelated detector

**DOI:** 10.1093/jmicro/dfae051

**Published:** 2024-11-07

**Authors:** Kousuke Ooe, Takehito Seki, Mitsuru Nogami, Yuichi Ikuhara, Naoya Shibata

**Affiliations:** Institute of Engineering Innovation, School of Engineering, The University of Tokyo, 2-11-16, Yayoi, Bunkyo, Tokyo 113-0032, Japan; Nanostructures Research Laboratory, Japan Fine Ceramics Center, 2-4-1, Mutsuno, Atsuta, Nagoya, Aichi 456-8587, Japan; Institute of Engineering Innovation, School of Engineering, The University of Tokyo, 2-11-16, Yayoi, Bunkyo, Tokyo 113-0032, Japan; PRESTO, Japan Science and Technology Agency, 4-1-8, Honcho, Kawaguchi, Saitama 332-0012, Japan; Institute of Engineering Innovation, School of Engineering, The University of Tokyo, 2-11-16, Yayoi, Bunkyo, Tokyo 113-0032, Japan; Institute of Engineering Innovation, School of Engineering, The University of Tokyo, 2-11-16, Yayoi, Bunkyo, Tokyo 113-0032, Japan; Nanostructures Research Laboratory, Japan Fine Ceramics Center, 2-4-1, Mutsuno, Atsuta, Nagoya, Aichi 456-8587, Japan; Institute of Engineering Innovation, School of Engineering, The University of Tokyo, 2-11-16, Yayoi, Bunkyo, Tokyo 113-0032, Japan; Nanostructures Research Laboratory, Japan Fine Ceramics Center, 2-4-1, Mutsuno, Atsuta, Nagoya, Aichi 456-8587, Japan; Quantum-Phase Electronics Center (QPEC), The University of Tokyo, 7-3-1, Hongo, Bunkyo, Tokyo 113-8656, Japan

**Keywords:** OBF STEM, Low-dose imaging, Pixelated detector

## Abstract

Optimum bright-field scanning transmission electron microscopy (OBF STEM) is a recently developed low-dose imaging technique that uses a segmented or pixelated detector. While we previously reported that OBF STEM with a segmented detector has a higher efficiency than conventional STEM techniques such as annular bright field (ABF), the imaging efficiency is expected to be further improved by using a pixelated detector. In this study, we adopted a pixelated detector for the OBF technique and investigated the imaging characteristics. Because OBF imaging is based on the thick weak phase object approximation (tWPOA), a non-zero crystalline sample thickness is considered in addition to the conventional WPOA, where the pixelated OBF method can be regarded as the theoretical extension of single side band (SSB) ptychography. Thus, we compared these two techniques via signal-to-noise ratio transfer functions (SNRTFs), multi-slice image simulations, and experiments, showing how the OBF technique can improve dose efficiency from the conventional WPOA-based ptychographic imaging.

## Teaser

The imaging characteristics of optimum bright-field (OBF) scanning transmission electron microscopy (STEM) technique using a pixelated detector was investigated. It was shown via systematic calculations and experimental demonstrations that OBF STEM using a pixelated detector can visualize atomic structures even for thicker samples efficiently at low-dose

## Introduction

Scanning transmission electron microscopy (STEM) is a powerful technique in the field of materials science because it can directly observe atomic structures of local defects such as interfaces and surfaces. However, one of the most difficult tasks in STEM imaging is the observation of beam-sensitive materials. Beam-sensitive materials (e.g. battery materials, porous materials, and organic materials) require low-dose condition for the observation to suppress irradiation damage. However, the low-dose condition results in a worse signal-to-noise ratio (SNR) of images owing to the significant shot noise. Thus, high-dose-efficient imaging techniques are required to minimize beam damage in STEM.

Conventionally, STEM has used annular-shaped single detectors such as annular dark-field (ADF) [1] and annular bright-field (ABF) imaging [2]. However, annular detectors collect only a fraction of the transmitted electrons; thus, the imaging efficiency is poor. Recently, segmented/pixelated detectors have been developed as new-generation STEM detectors, where almost all transmitted electrons are detected by multiple detection channels or pixels [3,4]. Using these technologies, much richer information about transmitted electrons along azimuthal and radial scattering angles can now be obtained, and new imaging techniques start to be used experimentally at atomic-resolution such as differential phase-contrast (DPC) [5,6] or electron ptychography [7]. For low-dose applications, we recently developed an optimum bright-field (OBF) STEM that obtains the highest SNR for a given segmented detector via dedicated complex frequency filtering [8]. OBF imaging with a segmented detector has approximately 70 times higher imaging efficiency than ABF STEM, and even oxygen atomic sites inside zeolite, which is a highly beam-sensitive porous material, can be clearly visualized under low-dose conditions [9]. Furthermore, in OBF STEM, real-time processing is available using a high-speed segmented detector system, which is very helpful for practical low-dose experiments. However, in the segmented detector, the detection area is much coarser than that of the pixelated detector, which leads to a poor resolution on the diffraction plane. Therefore, as shown in our previous study [8], the theoretical imaging efficiency of the OBF is decreased by approximately half. Thus, the OBF technique using a pixelated detector and the theoretical study of its imaging properties are of much importance for further low-dose applications.

In this study, we investigate the imaging characteristics of OBF STEM using a pixelated detector. First, we summarize the theoretical framework of the OBF STEM using a pixelated detector. Conventionally, single side band (SSB) ptychography has been developed as an in-focus electron ptychographic technique and applied to low-dose observations [10,11]. However, SSB ptychography assumes an infinitesimally thin object under weak phase-object approximation (WPOA). After the development of the aberration corrector, the depth of focus of the STEM probe was comparable to or less than a few nanometers, which is much smaller than the usual TEM sample thickness. Thus, even if the sample can be regarded as weak phase object, WPOA-based SSB ptychographic imaging is not always appropriate for atomic-resolution imaging. Recently, a thick WPOA (tWPOA) model was established in which the sample thickness was incorporated into the WPOA. The tWPOA model successfully explains the ABF imaging model robustly under multiple focus conditions [12]. Because the OBF method reconstructs a phase-contrast image using the tWPOA model, it is expected that the OBF will offer more accurate and efficient reconstruction. Second, the imaging efficiency was compared between SSB ptychography and OBF using SNR transfer functions (SNRTFs). In the tWPOA model, the phase-contrast transfer function (PCTF) can be defined as a contrast transfer function from thick samples, which is called an integrated PCTF (iPCTF) [12]. In addition, using the recently developed STEM noise evaluation theory, the noise level of arbitrary linear reconstruction techniques in STEM can be calculated [13]. Thus, SNRTFs were calculated by normalizing iPCTFs by the noise level, which can fairly compare the dose efficiency among different imaging techniques. The SNRTF shows a proportionality factor of the obtainable SNR as a function of spatial frequency, and the imaging method with a higher SNRTF can achieve a better SNR for the same electron dose and sample potential. In this study, we calculated the SNRTFs under several observation conditions and discussed the differences between the techniques based on the (t)WPOA model. Finally, we compared the simulated and experimental images obtained using the SSB ptychographic and OBF methods. We evaluated the imaging properties of the both techniques under low-dose conditions in the presence of dynamical scattering.

## Theoretical framework

In our previous work, the analytical derivation of the OBF approach was shown in a general form, including both pixelated and segmented detectors [8]. In this paper, we briefly summarize the OBF technique using a pixelated detector, focusing on the differences between this technique and SSB ptychography under the tWPOA model [12].

**Fig. 1.**
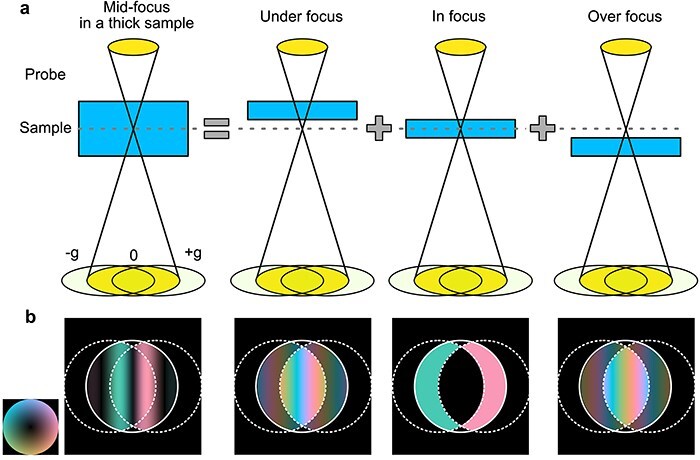
(a) Schematic illustration of the tWPOA model and (b) obtainable Fourier transformed 4D data $G\left(\mathbf k,\mathbf Q_\mathrm p\right)$, whose phase and amplitude are indicated with PAPUC color scheme [30]. In the tWPOA model, the diffraction intensity is given by the summation of scattered electrons from multiple thin slices, which can be treated as weak phase-object respectively.

In four dimensional (4D) STEM, the pixelated detector collects the transmitted electron intensity on the detector plane $\mathbf k$ at each probe position $\mathbf R_{\mathrm p}$, resulting in datasets $I\left(\mathbf k,\mathbf R_{\mathrm p}\right)$ as a function of $\mathbf{k}$ and $\mathbf R_\mathrm p$. In the OBF approach, the 4D data is Fourier transformed with respect to the probe position ${\mathbf R}_{\mathrm p}$. The obtained 4D data is described as $G\left(\mathbf k,{\mathbf Q}_{\mathrm p}\right)$ as a function of $\mathbf k$ and spatial frequency ${\mathbf Q}_{\mathrm p}$. Under the conventional WPOA, $G\left(\mathbf k,{\mathbf Q}_{\mathrm p}\right)$ is given as follows:


(1) $$\begin{aligned} G(\mathbf k,{\mathbf Q}_{\mathrm p})& ={\mathcal F}_{{\mathbf R}_{\mathrm p}\rightarrow{\mathbf Q}_{\mathrm p}}\lbrack I(\mathbf k,{\mathbf R}_{\mathrm p})\rbrack =\vert T(\mathbf k)\vert^2\delta({\mathbf Q}_{\mathrm p})+\mathrm i\lbrack T^\ast(\mathbf k)T(\mathbf k-{\mathbf Q}_{\mathrm p}) \nonumber \\ & -T(\mathbf k)T^\ast(\mathbf k+{\mathbf Q}_{\mathrm p})\rbrack\sigma V({\mathbf Q}_{\mathrm p}), \end{aligned}$$

where $T\left(\mathbf k\right)=A\left(\mathbf k\right)\exp\left(-\mathrm i\chi\left(\mathbf k\right)\right)$ is a lens transfer function whose modulus $A\left(\mathbf k\right)$ is an aperture function and $\chi\left(\mathbf k\right)$ is an aberration function. The term $T^\ast\left(\mathbf k\right)T\left(\mathbf k-\mathbf Q_\mathrm p\right)-T\left(\mathbf k\right)T^\ast\left(\mathbf k+ \mathbf Q_\mathrm p\right)$ describes interferences between a direct beam and scattered beams under the WPOA, giving non-zero values only in overlap areas between transmitted disk and single scattered disk (so-called double overlap region) if lens aberrations are perfectly corrected. In this case, this term only returns $ \mp 1$ for each double overlap region and zero everywhere else, working as a Fourier filter on $G\left(\mathbf k, \mathbf Q_\mathrm p\right)$ [14,15]. In SSB ptychography, phase component is reconstructed by collecting the double overlap regions of $G\left(\mathbf k,\mathbf Q_\mathrm p\right)$ at each $\mathbf Q_\mathrm p$, which is drawn as a schematic in Fig. 1(b) under in-focus condition or Fig. 3(c) with $\tau = 0$. If a phase plate or lens aberrations are present, phase modulation is introduced in $T\left(\mathbf k\right)$ and the regions other than the double overlap also have the non-zero value, which leads to more efficient reconstruction [16]. In the OBF technique, the phase-contrast component is reconstructed using the frequency filter $W\left(\mathbf k,\mathbf Q_\mathrm p\right)$, which is applied to all STEM images at each detector position $\mathbf k$. Considering the SNR at each Fourier component, which is obtained by normalizing the PCTF by noise-level at each $\mathbf Q_\mathrm p$, the most dose-efficient frequency filter $W\left(\mathbf k,\mathbf Q_\mathrm p\right)$ is derived as follows,


(2) $$\begin{aligned}W(\mathbf k,{\mathbf Q}_{\mathrm p})= \frac{K(\mathbf Q_\mathrm p)}{\mathrm i A^2_0} \lbrack T(\mathbf k)T^\ast(\mathbf k-{\mathbf Q}_{\mathrm p})-T^\ast(\mathbf k)T(\mathbf k+{\mathbf Q}_{\mathrm p})\rbrack.\end{aligned}$$

${A_0}$ is the area of probe-forming aperture in the reciprocal space and $K\left(\mathbf Q_\mathrm p\right)$ is a noise-normalization term, which makes the noise-level flat as a function of $\mathbf Q_\mathrm p$. The noise-normalization process can enhance contrast and resolution under low-dose condition [11,13]. By summing up the filtered 4D data on the detector plane as $\int W(\mathbf k, \mathbf Q_\mathrm p) G(\mathbf k, \mathbf Q_\mathrm p) \mathrm d \mathbf k$ and taking the Fourier transform of this, a real-space OBF image is obtained with a high dose-efficiency.

OBF reconstruction based on the WPOA model should be effective for thin samples below a few nanometers in thickness or two-dimensional materials. However, this approximation will break down in the case of thicker samples, even for weak phase specimens, because the electron propagation effect becomes non-negligible inside the crystals. Recently, the propagation effect is taken into account under WPOA, which is called the thick WPOA (tWPOA) model [12]. The OBF process can also adopt the tWPOA model as follows: In tWPOA, a thick sample is divided into a number of thin slices, and each slice is treated as a weak phase object. Each slice can be assumed to be identical for crystalline samples. Therefore, the exit wave is given as a superposition of transmitted and single-scattered electrons from each slice [12,17]. Thus, the $G\left(\mathbf k,\mathbf Q_\mathrm p\right)$ under tWPOA, denoted as $G^{\mathrm t\mathrm h\mathrm i\mathrm c\mathrm k}\left(\mathbf k,\mathbf Q_\mathrm p\right)$ in this study, is described as follows:


(3) $$\begin{aligned} G^\mathrm{thick}(\mathbf k,{\mathbf Q}_{\mathrm p})& =\vert T(\mathbf k)\vert^2\delta({\mathbf Q}_{\mathrm p})+\mathrm i\frac{\sigma V(\mathbf Q_\mathrm p)}{t} \int^{\Delta f + t}_{\Delta f}\lbrack T^\ast(\mathbf k)T(\mathbf k-{\mathbf Q}_{\mathrm p}) \nonumber \\ & -T(\mathbf k)T^\ast(\mathbf k+{\mathbf Q}_{\mathrm p})\rbrack \mathrm d z, \end{aligned}$$

where $t$ and ${{\Delta }}f$ are the sample thickness and defocus with reference to an entrance surface of the sample. The first and second terms correspond to the transmitted and scattered electrons from each slice in the thick sample, respectively, which is given by an integration along the thickness direction. Figure 1(a) shows the schematic of the tWPOA model. When the STEM probe is focused on the middle of the specimen (denoted as mid-focus condition), the obtainable $G^{\mathrm t\mathrm h\mathrm i\mathrm c\mathrm k}\left(\mathbf k,\mathbf Q_\mathrm p\right)$ is represented by the summation of $G\left(\mathbf k,\mathbf Q_\mathrm p\right)$ from in-focused plane and under/over focused planes. For simplicity, if we assume that all aberrations other than defocus are corrected as $\chi\left(\mathbf k;\Delta f\right)=\pi\lambda\Delta f\left|\mathbf k\right|^2$, $G\left(\mathbf k,\mathbf Q_\mathrm p\;\right)$ can be written as follows;


(4) $$ \begin{aligned} G(\mathbf k,{\mathbf Q}_{\mathrm p};\Delta f)& =\mathrm iA(\mathbf k)\lbrack A(\mathbf k-{\mathbf Q}_{\mathrm p})e^{-\mathrm i\chi(\mathbf k-{\mathbf Q}_{\mathrm p};\Delta f)+\mathrm i\chi(\mathbf k;\Delta f)}\\ & -A(\mathbf k+{\mathbf Q}_{\mathrm p})e^{\mathrm i\chi(\mathbf k+{\mathbf Q}_{\mathrm p};\Delta f)-\mathrm i\chi(\mathbf k;\Delta f)}\rbrack\sigma V({\mathbf Q}_{\mathrm p})\\ & =\mathrm iA(\mathbf k)\lbrack A(\mathbf k-{\mathbf Q}_{\mathrm p})e^{\mathrm i\pi\lambda\Delta f(2{\mathbf Q}_{\mathrm p}\cdot\mathbf k-\vert Q_{\mathrm p}\vert^2)})\\ & -A(\mathbf k+{\mathbf Q}_{\mathrm p})e^{\mathrm i\pi\lambda\Delta f(2{\mathbf Q}_{\mathrm p}\cdot\mathbf k+\vert Q_{\mathrm p}\vert^2)})\rbrack \sigma V({\mathbf Q}_{\mathrm p}). \end{aligned} $$

The in-focused plane is illuminated by the non-defocused probe (${{\Delta }}f = 0$), which results in the flat phase pattern in $G\left(\mathbf k,\mathbf Q_\mathrm p\right)$ as shown in Fig. 1(b). Contrastingly, the top and bottom planes are illuminated by the under- and over-focused probe respectively and $G\left(\mathbf k,\mathbf Q_\mathrm p\right)$ shows the phase variation inside the double/triple overlap regions, which is described as $e^{\mathrm i\pi\lambda\Delta f\left(2\mathbf Q_\mathrm p\cdot \mathbf k\mp\left|\mathbf Q_\mathrm p\right|^2\right)}$. In the case of the mid-focus condition, the under-/over-focused planes are symmetrically distributed with respect to the mid-plane and there are multiple pairs of two focal planes that have the same amplitude, but opposite sign defocuses. Because $G\left(\mathbf k,\mathbf Q_\mathrm p;-\Delta f\;\right)=G^\ast\left(\mathbf k,\mathbf Q_\mathrm p;\Delta f\;\right)$ from Eq. (4), imaginary part of the phase term $e^{\mathrm i\pi\lambda\Delta f\left(2\mathbf Q_\mathrm p\cdot \mathbf k\mp\left|\mathbf Q_\mathrm p\right|^2\right)}$ in $G\left(\mathbf k,\mathbf Q_\mathrm p;\Delta f\;\right)$ and $G\left(\mathbf k,\mathbf Q_\mathrm p;-\Delta f\;\right)$ is canceled out. Thus, the integration in Eq. (3) can be performed as follows:


(5) $$ \begin{aligned} G^{\mathrm{thick}}(\mathbf k,{\mathbf Q}_{\mathrm p})=& \vert T(\mathbf k)\vert^2\delta({\mathbf Q}_{\mathrm p})
+ \mathrm i A(\mathbf k)[\mathrm{sinc}(\omega_1 t) A(\mathbf k - {\mathbf Q}_{\mathrm p})\\ & -\mathrm{sinc}(\omega_2 t) A(\mathbf k + {\mathbf Q}_{\mathrm p})]\sigma V(\mathbf Q _\mathrm p), \end{aligned} $$

where ${\mathrm{sinc}}\left( x \right) = \sin \left( x \right)/x{\ }$ when $x \ne 0{\ }\left( {{\mathrm{sinc}}\left( 0 \right) = 1} \right),$  $\omega_1=\pi\lambda\left(\mathbf k\cdot \mathbf Q_\mathrm p-\left|\mathbf Q_\mathrm p\right|^2/2\right)$, and $\omega_2=-\pi\lambda\left(\mathbf k\cdot \mathbf Q_\mathrm p+\left|\mathbf Q_\mathrm p\right|^2/2\right)$. Eq. (5) indicates that the intensity is preserved after the integration on the lines given by $2\mathbf Q_\mathrm p\cdot \mathbf k\mp\left|\mathbf Q_\mathrm p\right|^2=0$ but decay with oscillation with the sinc function when apart from these lines. Thus, the intensity of $G^{\mathrm t\mathrm h\mathrm i\mathrm c\mathrm k}\left(\mathbf k,\mathbf Q_\mathrm p\;\right)$ has line-shaped intensity distribution as shown in the left-most panel in Fig. 1(b), which appears more clearly under larger thickness conditions. In the literature on SSB ptychography [18], similar lines are referred to as ‘acromatic lines’, which are shown to appear due to the defocus spread based on the conventional WPOA model (zero thickness). It is noted that the defocus variation inside the thick sample owing to the electron propagation and defocus spread for an infinitely thin sample, such as graphene, can be regarded as a very close condition, offering similar behavior to 4D datasets. Here, the obtained $G^{\mathrm t\mathrm h\mathrm i\mathrm c\mathrm k}\left(\mathbf k,\mathbf Q_\mathrm p\right)$ under the tWPOA model have non-zero values for not only double overlap region but also triple overlap region even with an aberration-free probe other than the defocus. Herein, the OBF filter for the tWPOA model $W^{\mathrm t\mathrm h\mathrm i\mathrm c\mathrm k}\left(\mathbf k,\mathbf Q_\mathrm p\right)$ is calculated by the same manner as Eq. (2);


(6) $$ \begin{aligned} W^{\mathrm{thick}}(\mathbf k,{\mathbf Q}_{\mathrm p})&=\frac{K^{\mathrm{thick}}(\mathbf Q_\mathrm p)}{\mathrm i A^2_0 t} \int_{\Delta f}^{\Delta f+t}\lbrack T(\mathbf k)T^\ast(\mathbf k-{\mathbf Q}_{\mathrm p})\\ & -T^\ast(\mathbf k)T(\mathbf k+{\mathbf Q}_{\mathrm p})\rbrack\mathrm dz. \end{aligned} $$

Thus, taking the inverse Fourier transform of $\int W^\mathrm{thick}(\mathbf k,{\mathbf Q}_{\mathrm p})G^\mathrm{thick}(\mathbf k,{\mathbf Q}_{\mathrm p})\mathrm d\mathbf k$, OBF image for the thick weak phase-objects is obtained. It is noted that OBF and other ptychographic techniques have been shown to obtain the highest contrast at the mid-plane condition [8,19,20], and we use this condition for the image simulation and experimental data acquisition. However, one can select other defocus condition such as surface focus condition and obtain the OBF image using the frequency filter $W^{\mathrm t\mathrm h\mathrm i\mathrm c\mathrm k}\left(\mathbf k,\mathbf Q_\mathrm p\right)$ that is dedicated for the corresponding focus condition. As for the term $K\left(\mathbf Q_\mathrm p\right)$ in Eq. (2) and $K^{\mathrm t\mathrm h\mathrm i\mathrm c\mathrm k}\left(\mathbf Q_\mathrm p\right)$ in Eq. (6), we can choose any shapes of Fourier filter. One may set this filter to normalize the noise level of reconstructed images, which improves the contrast and visibility under the low-dose conditions [8,11,13]. We also employ this scheme for the reconstruction, and detailed shape of noise-normalizing filter is given as follows:


(7) $$ \begin{aligned} K({\mathbf Q}_{\mathrm p})&=\left(A_0\int\left| T(\mathbf k)T^\ast(\mathbf k-{\mathbf Q}_{\mathrm p})-T^\ast(\mathbf k)T(\mathbf k+{\mathbf Q}_{\mathrm p})\right|^2\mathrm d\mathbf k\right)^{-1/2}\\ K^{\mathrm{thick}}({\mathbf Q}_{\mathrm p}) & =\left(A_0\int\left|\frac1t\int_{\Delta f}^{\Delta f+t}\lbrack T(\mathbf k)T^\ast(\mathbf k-{\mathbf Q}_{\mathrm p}) \right. \right. \\ & \quad \left.\left. \vphantom{\frac1t\int_{\Delta f}^{\Delta f+t}} -T^\ast(\mathbf k)T(\mathbf k+{\mathbf Q}_{\mathrm p})\rbrack\mathrm dz\right|^2\mathrm d\mathbf k\right)^{-1/2}. \end{aligned} $$

This is a straightforward extension of noise-normalizing filter used in OBF reconstruction via segmented detectors [8]. It is also mentioned that this OBF reconstruction scheme is based on the linear and non-iterative approach. Recently multi-slice ptychography, which adopts full dynamical multi-slice model for the ptychographic reconstruction, is intensively investigated particularly for thick samples [21]. Though this technique is also promising for the quantitative phase retrieval, it is based on the non-linear and iterative calculations, which is computationally expensive and sometimes leads to inappropriate results for interpreting the atomic structure [22]. While OBF approach does not include the multiple scattering effects, the non-iterative and linear reconstruction offers faster and reproducible results. In addition, OBF can be implemented as a real-time observation function [9], which should help users for experimental low-dose observations.

## Methods

STEM image simulations were conducted by the $\mu $STEM package [23], which is based on the multi-slice model to obtain 4D dataset. For the calculation of the STEM images and SNRTFs, all aberrations other than the defocus were assumed to be corrected. Observation experiments were performed using a JEM-ARM300F equipped with a pixelated detector (4DCanvas [4]). Using the four-fold binning acquisition mode of the 4Dcanvas detector, we collected STEM convergent-beam electron diffraction (CBED) patterns at a frame rate of 4000 fps, equivalent to a dwell time of 250 $\mu$s. We chose an SrTiO_3_ crystal with a [001] zone-axis as a model sample for the simulations and experiments. The TEM sample was fabricated via mechanical polishing and Ar ion milling.

## Results and Discussions

### Imaging efficiency under the tWPOA model

As described in the theoretical framework section, the OBF achieves the highest SNR under the tWPOA model and is expected to be more applicable for thicker samples. First, we examined the improvement in dose efficiency of the OBF technique compared with SSB ptychography, which relies on the conventional WPOA model.

**Fig. 2.**
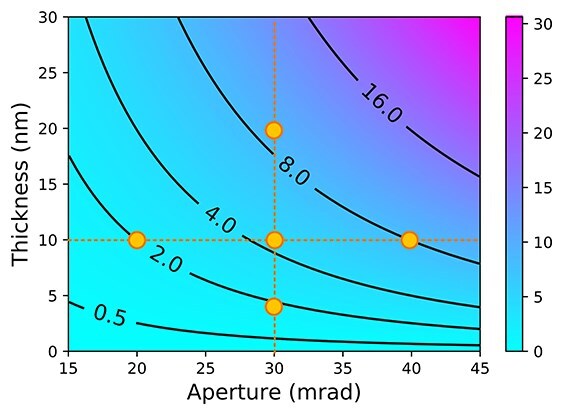
A counter map of normalized thickness $\tau $ as a function of sample thickness (nm) and probe-forming aperture angle (mrad) with an accelerating voltage of 300 kV. The orange dots indicate the conditions used for image simulations shown in Fig. 4.

**Fig. 3.**
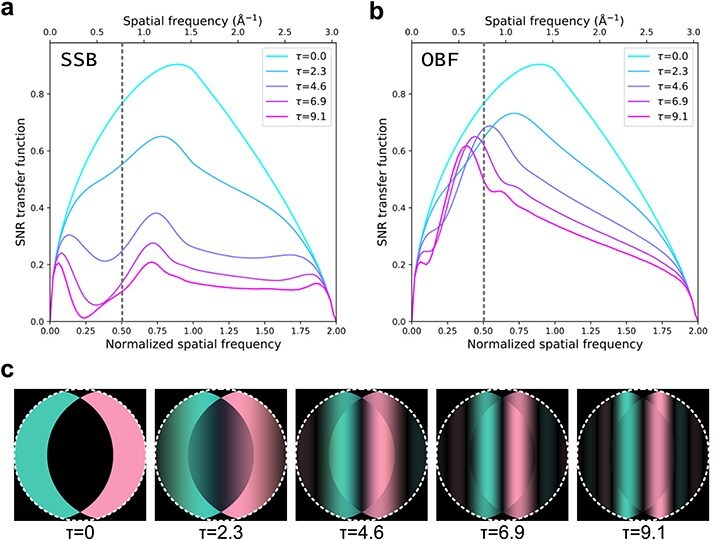
Comparison of SNRTFs of (a) SSB ptychography and (b) OBF under various thickness conditions. The thickness change is represented by the normalized thickness ${{\tau }}$, which is determined by the sample thickness, accelerating voltage, and convergence angle as shown in Fig. 2(c). The Fourier transformed 4D data $G\left(\mathbf k,\mathbf Q_\mathrm p\right)$ with several normalized thicknesses $\tau $. These $\tau $ values are the same as the SNRTF calculation shown in (a) and (b). The white dotted circle indicates the edge of the direct beam disk area.

As in the conventional WPOA model, the PCTF under the tWPOA model is also defined as the iPCTF, which shows contrast transfer from thick weak phase objects. Before the comparison, we introduce a dimensionless parameter $\tau = t/{{\Delta }}{z_p} = t\lambda k_0^2$, where ${{\Delta }}{z_p} = 1/\lambda k_0^2$, $\lambda $, and ${k_0}$ are the depth-of-focus [24], wavelength of electron beam determined by the accelerating voltage, and aperture radius on the reciprocal space, respectively. The parameter $\tau $ is calculated as a function of STEM optical conditions such as a sample thickness, an accelerating voltage, and a probe-forming aperture. $\tau $ is closely related to the inverse of the Fresnel number and serves as an indicator of when diffraction effects become significant. When $\tau \ll 1$, the thickness of the sample can be neglected, whereas when $\tau \approx 1$, it can no longer be ignored. The identical $\tau $ provides the identical iPCTFs as long as a defocus position is the same [8,25,26]. Therefore, we can calculate the SNRTFs and discuss the imaging properties in a generalized manner using this dimensionless parameter under tWPOA. Figure 2 shows a $\tau $ contour map as a function of sample-thickness and probe-forming aperture size with an accelerating voltage of 300 kV. This map indicates that the sample thickness against the probe depth focus increases with a physically larger sample thickness or larger convergence angle. In these cases, the propagation effect is expected to be substantial, even for weak phase objects, as shown in Fig. 1. Figures 3(a, b) show a comparison of SNRTFs between SSB ptychography and OBF for various $\tau $ values. For the small $\tau $ value, which is corresponding with a thin sample or large depth-of-focus case, SNRTFs of both techniques show similar results. Figure 3(c) shows the $G^{\mathrm t\mathrm h\mathrm i\mathrm c\mathrm k}\left(\mathbf k,\mathbf Q_\mathrm p\right)$ pattern calculated using the same $\tau $ values as those shown in Fig. 3(a, b). $G^{\mathrm t\mathrm h\mathrm i\mathrm c\mathrm k}\left(\mathbf k,\mathbf Q_\mathrm p\right)$ with $\tau = 0$ (infinitesimally thin sample) shows perfectly flat pattern inside the double overlap regions because the effects of beam propagation are minimal, and projection approximation holds. $G^{\mathrm t\mathrm h\mathrm i\mathrm c\mathrm k}\left(\mathbf k,\mathbf Q_\mathrm p\right)$ with $\tau $=2.3 (e.g. corresponding to accelerating voltage of 300 kV, 30 mrad probe forming aperture, and 5 nm thickness) shows the almost flat pattern though the triple overlap region has slight non-zero values. Therefore, with the small $\tau $ value, difference between conventional WPOA and tWPOA is relatively small and SNRTFs of SSB and OBF are also alike. However, this difference becomes substantial with higher $\tau $ value (thicker sample, larger convergence angle, or higher accelerating voltage) as shown Fig. 3. For instance, when $\tau $ is 4.6 (e.g. accelerating voltage of 300 kV, 30 mrad probe forming aperture, and 10 nm thickness) the SNRTF of SSB ptychography gets significantly worse compared with that of OBF. This is because contribution to the reconstructed image from the slices illuminated by the defocused probe regions gets larger and larger with higher $\tau $ value, which is not considered in SSB ptychography but done in OBF. In this case, the amplitude of $G^{\mathrm t\mathrm h\mathrm i\mathrm c\mathrm k}\left(\mathbf k,\mathbf Q_\mathrm p\right)$ on the triple overlap region becomes dominant. Furthermore, the amplitude in double overlap regions almost disappeared with $\tau $ of 9.1. SSB ptychography takes filtering and integration of $G^{\mathrm t\mathrm h\mathrm i\mathrm c\mathrm k}\left(\mathbf k,\mathbf Q_\mathrm p\right)$ only inside the double overlap regions and zeroing out everywhere else because only these regions are assumed to have the amplitude in the conventional WPOA model. However, this is outside of the range of WPOA model validity and results in the significantly worse SNRTF by missing the triple-overlap regions for large $\tau $ cases. On the other hand, the SNRTF of OBF retains its dose-efficiency for larger $\tau $ since OBF uses both signals in double and triple overlap regions based on the tWPOA model. Therefore, a higher dose efficiency of the OBF technique is expected for thicker specimens.

**Fig. 4.**
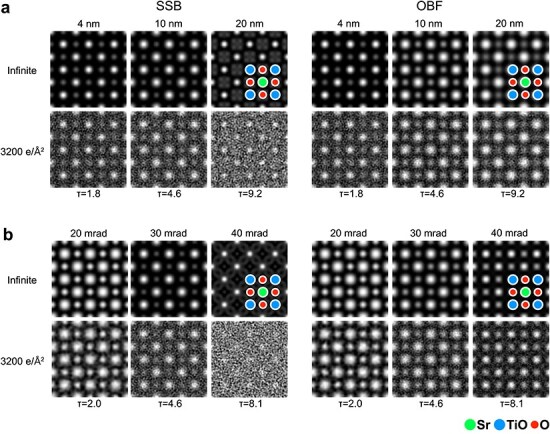
(a) Thickness series and (b) probe-forming aperture series of simulated SSB ptychographic and OBF images at an accelerating voltage of 300 kV. An aperture size of 30 mrad was assumed for the thickness series simulations and a sample thickness of 10 nm was assumed for the aperture size series simulations. For the electron dose, an infinite dose (noise free) and 3200 e/Å^2^ were assumed. The $\tau $ values assumed for the simulation are also shown.

### OBF image simulation by multi-slice calculation

To examine the imaging efficiency of the OBF technique, we performed a multi-slice image simulation of the SrTiO_3_ [001] sample. Figure 4 shows the simulated SSB ptychography and OBF images for various thicknesses (Fig. 4(a)) and probe-forming apertures (Fig. 4(b)). Under each condition, we calculated images with infinite dose conditions (noise-free) and finite dose conditions (assumed Poisson noise with an electron dose of 3200 e/Å^2^). In the thickness-series shown in Fig. 4(a), the simulated SSB and OBF images with $\tau $ = 1.8 are very similar under not only the noise-free case but the finite dose condition because the normalized sample thickness is sufficiently small as expected from the SNRTFs shown in Fig. 3. However, in the case of $\tau $ = 4.6 (10 nm thickness with 30 mrad aperture), OBF visualizes atomic structures more clearly under the finite dose condition than SSB ptychography. While the SSB ptychographic image shows proper contrast with infinite dose, it offers a worse SNR with a finite dose than the OBF. In this case, the $\tau $ is 4.6 and the propagation effect gets non-negligible as shown in the SNRTF calculation (Fig. 3). Therefore, it can be said that OBF imaging based on the tWPOA model is more dose-efficient for thicker samples even in the presence of dynamical scattering effect. Furthermore, the SSB ptychographic image with 20 nm sample thickness no longer provides proper contrast at the oxygen site and shows a much worse SNR in the case of a finite dose. As shown in Fig. 3, the SNRTF of SSB ptychography starts to oscillate at the larger $\tau $ values and it has much lower value at some spatial frequency domains, which could lead to worse visibility of oxygen sites. On the other hand, the OBF image-contrast still visualizes atomic structures well, including both heavy and light element sites, under both infinite and low-dose conditions. Thus, it is shown that OBF has the capability of dose-efficient imaging even with thicker samples, such as 20 nm. Next, in the probe-forming aperture series shown in Fig. 4(b), similar imaging properties are obtained as well as the thickness-series results discussed above. With a 20 mrad aperture, 300 kV voltage, and 10 nm sample thickness, the corresponding $\tau $ is 2.0. The SSB and OBF images show similar contrast under both (in)finite dose conditions because the 10 nm sample can be regarded as ‘thin’ against the depth-of-focus of 20 mrad aperture according to the $\tau $ value. However, if the aperture becomes large as 40 mrad, the $\tau $ gets 8.1 because the depth-of-focus significantly gets smaller with larger apertures [27]. This situation also leads to the breakdown of the conventional WPOA model, and SSB ptychographic images do not offer the proper contrast and dose efficiency. In this case, the OBF can visualize SrTiO_3_ atomic sites with a higher SNR, showing the capability to observe atomic structures with a large convergence angle. By these systematical simulations (thickness-series and aperture-series), it is noted that the tWPOA model and dimensionless parameter $\tau $ are helpful to understand imaging properties of these techniques.

**Fig. 5.**
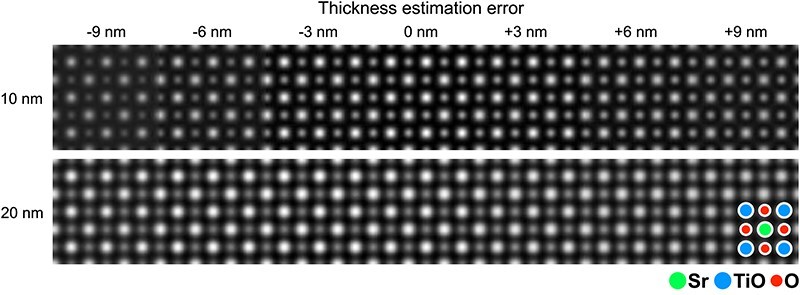
Simulated OBF images with true thicknesses (10 nm and 20 nm) and multiple erroneous estimated thickness parameters used for the OBF reconstruction (from −9 nm to +9 nm).

Furthermore, we also investigated effects of error for the thickness values used for the calculation of OBF reconstruction filter $W^{\mathrm t\mathrm h\mathrm i\mathrm c\mathrm k}\left(\mathbf k,\mathbf Q_\mathrm p\right)$. In practice, the accurate estimation of sample thickness is often difficult particularly if the crystalline structure of sample is unknown, which results in an error of the input thickness parameter for the OBF reconstruction. For the simulated 4D STEM datasets with thicknesses of 10 nm and 20 nm, we reconstructed multiple OBF images with different input thickness parameters as estimated values to calculate OBF filter shown in Eq. (6). For both thickness cases, the OBF image-contrast was maximized when the estimated thickness was equal to the true thickness that was used for the simulation as shown in Fig. 5. This indicates that we can estimate the sample thickness by maximizing the OBF image contrast as a function of input thickness parameters. It is also mentioned that the OBF image shows proper contrast to interpret atomic structures even if the estimated thickness was deviated from the true values. This holds for thicker samples such as 20 nm thickness, which should be helpful for the practical experiments to analyze the atomic structures.

### Experimental demonstration of low-dose OBF imaging with a pixelated detector

**Fig. 6.**
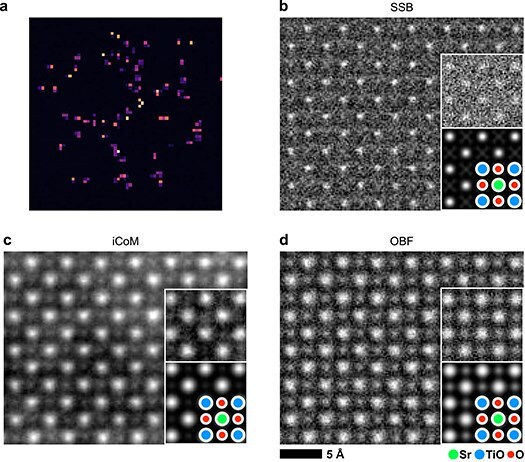
(a) A single frame of experimentally acquired 4D data $I\left(\mathbf k,\mathbf R_\mathrm p\right)$ from SrTiO_3_ [001] zone-axis. (b) Reconstructed SSB ptychographic image, (c) iCoM image, and (d) OBF image. All images were reconstructed from the same 4D data. The insets are the simulated images of each technique under the same observation conditions as the experiment. As for the electron dose in the simulated images, an infinite dose (noise-free) condition and a finite dose (same as the experiment) were assumed.

Finally, we conducted an experimental low-dose observation of SrTiO_3_ [001] using a pixelated detector 4DCanvas [4]. The 4DCanvas can read-out 4D data at 4000 fps with four-fold binning mode, resulting in dwell time of 250 $\mu$s/pixel. The sample thickness of the observed area was estimated to be 16 nm using the position-averaged CBED (PACBED) pattern [28], which resulted in a nonnegligible propagation effect, as shown in Fig. 4(a). This thickness corresponds to $\tau $=7.3 with an accelerating voltage of 300 kV and a convergence angle of 30 mrad, where the difference between SSB ptychography and OBF should be substantial. In this study, we used 4DCanvas in single-electron mode under low-dose conditions of 1250 e/Å^2^. Figure 6(a) shows an example of a single frame of the acquired 4D data, where the bright spots correspond to the detected electrons. Next, we reconstructed the SSB ptychographic and OBF images from the same dataset. In the SSB image shown in Fig. 6(b), the SNR is poor, and only heavy atomic sites are slightly visualized (Sr and Ti-O sites). We simulated the SSB ptychographic image under the same observation conditions, showing that the experimental and simulated images match well and that oxygen sites cannot be seen even under the infinite dose condition. As shown in Fig. 6(c), we also reconstructed an integrated center-of-mass (iCoM) image, which is utilized for light element and low-dose imaging in 4D STEM techniques as well as SSB ptychography [29]. Though the iCoM image visualizes oxygen sites better than the SSB ptychographic image, there is long range contrast fluctuation in the background resulted from an integration process of the original CoM images, which makes the visibility of atoms lower than OBF [9]. In contrast, as shown in Fig. 6(d), the OBF image reconstructed from the same dataset clearly shows the atomic structure of all atomic sites, which agrees well with the simulated image. Thus, we successfully visualized the atomic structures of thicker SrTiO_3_ [001] samples at lower dose, experimentally demonstrating the capability of low-dose OBF imaging using a pixelated detector.

## Concluding remarks

The OBF STEM technique using a pixelated detector was studied based on the thick WPOA (tWPOA) model, in which the propagation effect inside a thick sample was considered in addition to the conventional WPOA. The OBF technique has a much higher dose efficiency than SSB ptychography, particularly for thicker samples or larger probe-forming apertures. This effectiveness was confirmed by multi-slice simulations and experiments using a pixelated detector, even in cases of appreciable dynamical scattering, offering more reliable atomic structure observations under low-dose conditions. Thus, OBF STEM using a pixelated detector is a promising technique for low-dose experiments, particularly for thicker samples.

## Abbreviations

OBF: Optimum Bright-Field STEM: Scanning Transmission Electron Microscopy (t)WPOA: (thick) Weak Phase Object Approximation SSB: Single Side Band SNRTF: Signal-to-noise Ratio Transfer Function
